# Genomic sequencing of vineyard-isolated *Bacillus* strains and comparison with closely related *Bacillus* species reveals a putative new lineage, distinct pan-genome architecture and secondary metabolism potential

**DOI:** 10.3389/fmicb.2026.1871298

**Published:** 2026-08-13

**Authors:** Adeoye J. Kayode, Edwin I. Wanjofu, Mathabatha Evodia Setati

**Affiliations:** 1 South African Grape and Wine Research Institute, Stellenbosch University, Private Bag X1, Stellenbosch, Western Cape province, South Africa; 2 Department of Biochemistry, Genetics and Microbiology, University of Pretoria, Pretoria, South Africa

**Keywords:** *Bacillus* spp., biocontrol, bunch rot, secondary metabolites, whole genome sequencing

## Abstract

**Introduction:**

*Bacillus* species are ubiquitous in nature, and they display diverse traits of biotechnological importance, including the production of various enzymes and secondary metabolites. In this study, we profiled the genomes of vineyard-derived *Bacillus* strains shown to inhibit *Botrytis cinerea* mycelial growth and spore germination through the production of cyclic lipopeptides and cell wall-degrading enzymes.

**Methodology:**

Long-reads-whole genome sequencing was conducted using PacBio Sequel II Single-Molecule Real-Time platform, followed by genome assembly, phylogenetic analysis and functional annotation using different bioinformatics pipelines.

**Results:**

The strains possess ~3.900,000 bp genome size and %GC ranging between 45%–46.7%, as expected in *Bacillus* species. Phylogenetic analysis separated the vineyard-derived strains into two lineages. Strains B4003 and B4005 grouped with *B. velezensis*. Notably, three *Bacillus* strains (B4022, B4001, and B4023) clustered with the undescribed strains with placeholders “sp018613535”, an undescribed taxon in the genome taxonomy database, supported by the Average Nucleotide Identity (ANI) score above 96%. Putative biosynthetic gene clusters (BGCs) involved in secondary metabolite production were identified by AntiSMASH analysis among the isolates. Orthologous cluster analysis further identified 3203 genes conserved across all strains, constituting a stable core genome that supports essential cellular and metabolic functions. Comparative pan-genome analysis of the *Bacillus velezensis* and *Bacillus* spp. revealed clear species-level differentiation alongside a conserved functional backbone. Furthermore, functional annotation revealed strain-specific enrichment in metabolic and environmental response pathways, emphasizing the ecological specialization and adaptive diversification.

**Discussion:**

Overall, these findings emphasize the dynamic interplay between genomic conservation and flexibility within the *Bacillus* genus and support the biological control potential of these strains. Furthermore, genomic evidence strongly indicates that B4001, B4022 and B4023, represent members of a putatively novel species.

## 1 Introduction

The genus *Bacillus* is classified in the phylum Firmicutes and comprises 435 species organized into two major clades: the Subtilis clade and the Cereus clade (Bhandari et al., 2013; Blanco Crivelli et al., 2024). *Bacillus* species are ubiquitous in nature and thrive under varying climatic and edaphic conditions. Moreover, a plethora of studies have demonstrated that this genus exhibits substantial taxonomic, genetic and metabolic diversity. Indeed, not only are *Bacillus* species proven to produce different secondary metabolites (e.g., lipopeptides, enzymes, vitamins, and platform chemicals), but some also display plant growth promotion properties as well as biological control against many phytopathogens, while others are used as hosts for heterologous gene expression. Species across both the *B. subtilis* clade, which includes *B. subtilis, B. licheniformis, B. pumilus* and *B. amyloliquefaciens*, and the *B. cereus* clade, which includes *B. anthracis, B. cereus* and *B. thuringiensis*, have gained considerable interest in agriculture due to their potential in disease management and enhancing agricultural productivity (Bhandari et al., 2013; Blanco Crivelli et al., 2024; Cai et al., 2017; Calvo et al., 2021; Collinge et al., 2022). Due to their diverse secondary metabolism, many of the species have been documented to produce valuable bioactive compounds with broad-spectrum activity against plant pathogens (Wang et al., 2023; Zhao et al., 2025). For instance, *Bacillus* spp. can produce nonribosomal peptides such as bacillibactin, bacillomycin D, fengycin, and surfactin, polyketides such as macrolactin, difficidin, and bacillaene (Put et al., 2024; Shan et al., 2025; Zaid et al., 2022; Zeng et al., 2021), as well as lytic enzymes that can break down phytopathogen cell walls and decompose complex substrates for nutrient uptake (Jin et al., 2025; Singh et al., 2025). Furthermore, the plant growth-promoting (PGP) attributes of *Bacillus* strains, such as growth stimulation or modulation of phytohormones (indole-3-acetic acid, IAA), nutrient uptake enhancement (phosphate solubilization and nitrogen fixation), volatile compounds emission (2,3-butanediol) and systemic resistance enhancement have been documented (Pal et al., 2022).

Over the past decade, there has been a growing interest in either replacing or supplementing chemical fungicides with biological control agents (BCAs). BCAs can induce enhanced resistance against a pathogen, compete for space and nutrients with pathogens, secrete antimicrobials that damage the pathogen cell wall integrity and alter membrane permeability, inhibit pathogen mycelial growth and spore germination (Boro et al., 2022; Herrera-Défaz et al., 2023; Sheoran et al., 2025). While biological control capabilities have been demonstrated in several species, particularly within the *B. subtilis* clade, only *B. subtilis, B. licheniformis, B. amyloliquefaciens*, and *B. velezensis* have been extensively studied and therefore account for most of the commercial *Bacillus*-based BCAs. Other recently described species, *B. nakamurai* and *B. siamensis*, which together with *B. velezensis* belong to the *B. amyloliquefaciens* operational group, have also emerged as desirable BCAs. In our previous study, we reported several vineyard-derived *Bacillus* spp and strains that exhibited antifungal activity against different strains of *Botrytis cinerea*, a necrotrophic plant pathogen known to cause rot in many fruits, including grapes, leading to extensive economic losses. The isolates identified as strains of *B. velezensis, B. nakamurai, B. subtilis*, and *B. wiedmannii* through 16S rRNA, *rpo*B, *gyr*B, and *rec*A gene sequencing displayed varying abilities to inhibit *B. cinerea* mycelial growth and spore germination. Inter- and intraspecific variations in *B. cinerea* inhibition potency were partly attributed to the differences in the production of lytic enzymes and lipopeptide composition profiles (Moremi et al., 2025).

While the antagonistic mechanisms of *Bacillus* spp. have been investigated, further studies are required to fully understand their biocontrol potential. Genetic studies involving genome sequencing, annotation and comparative analysis are crucial for advancing our understanding of the biology of the fungal antagonist. Distinguishing some strains of *Bacillus* spp. is seldom challenging; hence, several reclassifications have been done (Dunlap et al., 2016; Fan et al., 2017). Classifications based on traditional biochemical and physiological methods and phylogenetic analysis based on the 16S rRNA gene have proved inadequate for differentiating the strains. This study, therefore, sought to explore the taxonomy using whole-genome classification, comparatively explore the genetic composition and the biosynthetic inventory of vineyard-isolated *Bacillus* species and strains to unveil their potential as a biological control agent against *B. cinerea*.

## 2 Materials and methods

### 2.1 Bacterial strains

Five bacterial strains previously isolated from vineyard soil and identified as *B. velezensis* strain B4003 and B4005, as well as *Bacillus* spp. B4001, B4022 and B4023 were preserved in 25% (v/v) glycerol at −80 °C in the culture collection unit of the South African Grape and Wine Research Institute (SAGWRI), Stellenbosch University. These strains were revived by streaking on Nutrient Agar and incubating for 24 h at 30°C. Single colonies were transferred onto fresh agar and further used for genomic DNA extraction once sufficient growth was achieved.

### 2.2 Whole-genome sequencing

#### 2.2.1 Genomic DNA extraction

High-molecular-weight genomic DNA was extracted from overnight cultures using the Nanobind HMW DNA extraction kit following the procedure stipulated by the manufacturer. The DNA was quantified using the Qubit HS dsDNA to determine DNA concentration and checked for purity using the Nanodrop. DNA integrity and fragment-size distribution were assessed using the Agilent Tapestation system with genomic ScreenTape (Agilent Technologies, Santa Clara, CA, USA). Samples that passed QC (yield ≥3 μg, 260/280 ~ 1.8, 260/230 ~ 1.2–1.8, modal fragment > 15–20 kb) were accepted for HIFI library preparation. The average fragment size was found to be approximately 7–10 kb, indicating that no additional shearing was necessary. When short fragments were present, size selection (e.g., SRE) was applied.

#### 2.2.2 Library preparation

Bacteria genomes were sequenced on the PacBio^®^ Sequel II Single-Molecule Real-Time (SMRT) platform at Inqaba Biotechnical Industries (Pty) Ltd., South Africa. SMRTbell library was prepared using the SMRTlink guided protocol according to PacBio's Microbial Multiplexing workflow, adhering to the guidelines in PacBio's SMRTbell Prep Kit 3.0 procedure checklist for whole-genome libraries on the Sequel II system. The resulting SMRTbell libraries were re-quantified using the Qubit HS dsDNA Assay and checked for library quality on the TapeStation with Genomic DNA ScreenTape to ensure proper fragment size and library integrity prior to sequencing. The library binding step was conducted using Binding Kit 3.2, with Control 1.0 added according to PacBio's protocol. Sequencing primers and DNA polymerase were bonded with the Binding Kit 3.2. Control DNA (Control 1.0) was added to monitor sequencing performance. The library was loaded onto a Sequel II Sequencing Plate 2.0 after preparation. Sequencing was subsequently executed on the Sequel II system using the SMRT Cell 8M flow cell chip. The generated PacBio Sequel II subreads were processed using SMRT Link v11.1 (Pacific Biosciences, Menlo Park, CA, USA), employing the Circular Consensus Sequencing (CCS) algorithm to generate high-fidelity (HiFi) reads with predicted quality score >QV40.

### 2.3 Genome assembly and quality assessment

Briefly, raw PacBio subreads were imported into SMRT Link v11.1 (Pacific Biosciences, Menlo Park, CA, USA), where sequencing run quality was assessed using the data management module. Quality metrics including read length distributions, quality scores, sequencing yield and the N50 values for to assess sequencing performance. Raw subreads were subsequently processed using Circular Consensus Sequencing (CCS) algorithm implemented within the SMRT Link v11.1 to generate highly accurate HiFi reads (quality value >Q40). The CCS algorithm generated consensus sequences by aligning multiple subread passes around each circularized SMRTbell^®^-circularized DNA molecule, using the Quiver framework for error correction and consensus calculation. During this process, low-quality subreads, adapter and SMRTbell sequences, as well as poor-quality regions, were automatically trimmed, and reads below the predicted accuracy threshold were excluded (Wenger et al., 2019). Individual samples generated between 0.9 and 1.3 Gb of sequencing data, with mean read lengths ranging from 7.1to 11.1 kb (Supplementary Table 1. Approximately 582.98 Gb of polymerase read bases were generated from 6,212,824 polymerase reads with a mean polymerase read length of 93.8 kb, a polymerase read across the sequencing run.

Genomes were *De novo* assembled using the Circular Consensus Sequencing (CCS) algorithm implemented within the SMRT Link v11.1 with default parameters using the HiFi reads as input. Subsequently, the resulting assembly was polished and rotated using the Microbial Genome Analysis module within SMRT Link. Polishing was performed with the Racon algorithm (https://github.com/isovic/racon) (Vaser et al., 2017) and then OriC rotation is applied to produce the polished and rotated genome assemblies suitable for downstream analyses. The Microbial Genome Analysis module was also used to detect the N6-methyladenine (6mA) and N4-methylcytosine (4mC) modified bases and associated DNA sequence motifs based on based on sequencing kinetic information.

#### 2.3.1 Genome assembly quality and annotation

The assembled genomes' quality was then evaluated using Quast v5.3.0 (Gurevich et al., 2013) and checkM v1.0.18 (Parks et al., 2015). The genomes were then annotated with Prokka v1.14.6 with a “–proteins” flag, whereby the. *gbff* file of *Bacillus velezensis* FZB42 (accession: CP000560.2) for unified CDS nomenclature (Seemann, 2014).

### 2.4 Phylogenetic analyses and average nucleotide identity

The genomes sequenced in this study were queried against the Genome Taxonomy Database (GTDB) (Parks et al., 2022) through Kbase (Arkin et al., 2018). Based on the GTDB output, two genomes belonging to *Bacillus velezensis* and all the genomes belonging to *Bacillus* sp018613535 (which were all the genomes grouping with some of our genomes) were downloaded from NCBI. The *Bacillus* sensu stricto type genomes, according to the classification in the GTDB (accessed in September 2025) and LPSN (https://lpsn.dsmz.de/), were also downloaded from NCBI. The *Stenotrophomonas maltophilia* NCTC10257 genome was also downloaded for phylogenomics purposes as an outgroup. The phylogenomic analysis on the above set of genomes was achieved through the use of the Up-to-date Bacterial Core Gene (UBCG) pipeline (Kim et al., 2021; Na et al., 2018). The UBCG pipeline follows a series of predictions and extraction of 92 core genes in bacteria using Prodigal, which were then clustered into phylogenies using FastTree (Price et al., 2010). This generates individual gene phylogenies that were later used to construct a general phylogeny, which has the gene support index (GSI), where every branch represents how many gene trees support that branching (Na et al., 2018). Based on the UBCG phylogeny, the close relatives and the genomes conspecific to our strains, based on the GTDB, were used for the average nucleotide identity (ANI) calculations. The ANI was calculated using jspecies (https://jspecies.ribohost.com/jspeciesws/#analyse) employing the ANIb (blast) algorithm (Richter et al., 2016).

### 2.5 Pan-genome and orthologs analysis

The pangenome analysis of these strains was done using Roary v3.13.0 (Page et al., 2015), run at a 80% similarity boundary The blast similarity threshold was lowered to accommodate comparative genomics between the two species studied. The strains sequenced in this study, together with the additional conspecific strains from the GTDB database as described earlier, were included in the Pangenome analyses for robustness. The presence/absence matrix was then plotted into a heatmap using R programming. The genes shared among the strains isolated in this study were compared to each other in OrthoVenn3 (https://orthovenn3.bioinfotoolkits.net/home) using the Orthofinder ortholog clustering algorithm.

### 2.6 Functional characterization

The amino acid files of the individual strains were submitted to GhostKOALA (https://www.kegg.jp/ghostkoala/) for KEGG Orthology (KO) search and assignment (Aramaki et al., 2020) and the data were plotted out as relative numbers of the functional families predicted. The secondary metabolite analysis shell (antiSMASH–V8.0.4) resource was employed for the automatic genomic identification and analysis of biosynthesis gene clusters (BGCs) using the default detection parameters. Comparisons against biosynthetic gene clusters that have been experimentally characterized was achieved using the knownClusterBlast module and the MIBiG database, as provided by the default antiSMASH analysis workflow (Chen et al., 2018; Medema et al., 2011; Weber et al., 2015).

## 3 Results

### 3.1 Genomic features of the analyzed strains

Good-quality genomes were recovered from all the strains sequenced in this study. Individual samples yielded between 0.90 and 1.32 Gb of sequenced data, with mean read lengths ranging from 7.10 to 8.65 kb. They all had high genome coverages of between 225.8**×** and 336.6**×**, which is above the set threshold ideal for phylogenetic inferences (Chun et al., 2018). The genome assemblies were highly contiguous, and the contigs observed in each of the five strains ranged between one and four contigs. Almost the entire genomes were captured in a single dominant contig ~ 3.9 000,000 bp, which comprises most of the assembled sequence considered to represent the chromosomal backbone (Table 1). The remaining smaller contigs may represent plasmids or unresolved assembly fragments; however, we did not further investigate their identity in this study. Although the assemblies are highly contiguous and near complete, the presence of the predicted origin of replication (OriC) alone was not interpreted as evidence of genome circularity. We therefore describe the genome data according to the assembly status rather than complete circular genomes. In addition, the assembled genomes all possessed high N50 ranging from 3,876,429 to 3,971,905 bp, while they all have an L50 value of 1, indicating that at least 50% of each genome's assembly was contained within a single contig and < 2% contamination (above 98% completeness). As expected in *Bacillus* species, they all had a %GC between 45%–46.7%, with the strains conspecific to *Bacillus velezensis* (strains B4003 and B4005) having a high %GC of 46.53 and 46.65, respectively and circular chromosomes.

**Table 1:** Genome assembly quality metrices of PacBio HiFi.

Assembly quality metrics	B4022	B4003	B4005	B4001	B4023
Marker lineage	*Bacillus*	*Bacillus*	*Bacillus*	*Bacillus*	*Bacillus*
Reference genomes	36	36	36	36	36
Number of markers	1,200	1,200	1,200	1,200	1,200
Marker sets	269	269	269	269	269
Marker genes detected ones	1,189	1,189	1,189	1,189	1,189
Marker genes detected twice	4	6	1	6	4
Missing marker genes	7	1	1	6	7
Genome Size (bp)	3,996,852	3,964,064	3,916,708	3,925,055	3,988,914
Bases generated (Gb)	1.041	0.905	1.318	1.273	0.901
Mean read length (kb)	7/863	7.642	8.652	7.101	7.374
Coverage (**×**)	260.3	228.4	336.6	324.3	225.8
Contigs	3	3	1	4	3
Maximum contig length	3,971,597	3,876,429	3,914,230	3,885,103	3,971,905
N50 contig length	3,971,597	3,876,429	3,914,230	3,885,103	3,971,905
L50	1	1	1	1	1
Sum of contig lengths	3,995,053	3,964,064	3,914,230	3,922,880	3,986,339
E-size (sum of squares/sum)	3,948,362	3,792,226	3,914,230	3,847,816	3,957,550
Completeness	98.43	99.81	99.81	98.62	98.43
Contamination	1.05	0.05	0.05	1.29	1.05

Genome completeness and contamination were evaluated using CheckM. Sequencing depth was estimated by dividing the total number of bases generated for each sample by the corresponding assembled genome size.

Critically, the highest coding genes (3,928) were observed in *Bacillus* sp. strain B4022, followed by 3916 for *Bacillus* sp. strain B4023. Similarly, predicted genes were more abundant in *Bacillus* sp. strain B4022, followed by for *Bacillus* sp. strain B4023. However, higher numbers of pseudogenes (24 pseudogenes, respectively) were detected in these strains. The total predicted RNAs ranged from 131 to 136, with 137 RNAs found in strain *Bacillus* sp. strain B4001, followed by *Bacillus* sp. strain B4022. Among the RNAs (rRNAs and tRNAs, tmRNAs, ncRNAs) predicted, tRNAs were the most abundant, with either 86 or 87 tRNAs found in each strain, followed by rRNAs (27 each) and ncRNAs. Hypothetical proteins ranged from 97–212, and *Bacillus* sp. strains B4022 and B4023 had the highest predicted (212 and 205, respectively while 97, 118 and 157 were found in *B. velezensis* strains B4003, B4005 and B4001, respectively. Genomic elements comprising sORFs, oriCs, and oriVs were predicted, with 4 sORFs each detected in all strains, 2 oriCs and oriVs each found in all strains except for *B. velezensis* strains (B4003 and B4005) that lack oriVs (Table 2).

**Table 2:** Genome features of vineyard-isolated *Bacillus* strains.

Genome properties	B4022	B4003	B4005	B4001	B4023
Genome Size (bp)	3,996,852	3,964,064	3,916,708	3,925,055	3,988,914
Contigs	3	3	1	4	3
G + C content (%)	45.29	46.53	46.65	45.32	45.3
Predicted genes	4,045	3,888	3,858	3,914	4,033
Coding genes	3,928	3,774	3,744	3,799	3,916
Pseudogenes	24	10	13	10	24
Coding density	89.3	90.1	90.2	89.1	89.3
RNAs	136	132	131	137	135
tRNAs	87	86	86	86	87
tmRNAs	1	1	1	1	1
rRNAs	27	27	27	27	27
ncRNAs	21	18	17	23	20
ncRNA regions	60	59	60	60	60
Hypotheticals	212	97	118	157	205
sORFs	4	4	3	4	4
oriCs	2	2	2	2	2
oriVs	2	-	-	2	2

tRNA, transfer RNAs; tmRNAs, transfer-messenger RNAs; rRNAs, ribosomal RNAs; ncRNAs, non-coding RNAs; sORFs, short open reading frames; oriCs, replication origins; oriVs, vegetative replication origins.

### 3.2 Phylogenetic inferences and Average Nucleotide Identity (ANI)

**Figure 1:**
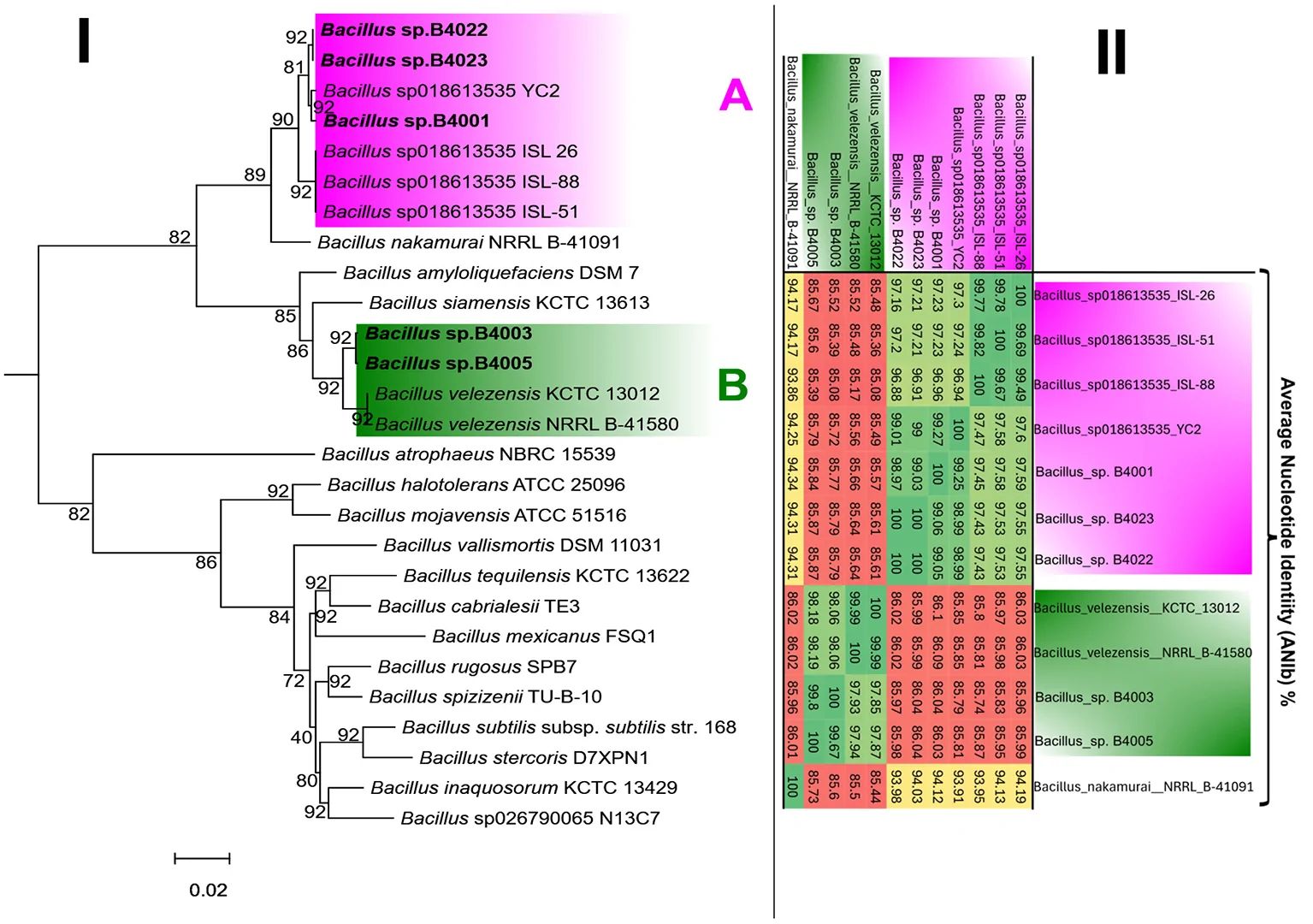
**(I)** Genome-based phylogenetic subtree of *Bacillus* spp. constructed using the UBCG pipeline, incorporating reference genomes retrieved from the GTDB database. Numbers on the nodes represent the Gene Support Index (GSI). The complete phylogeny is shown in Supplementary Figure 1 of the Supplementary material. **(II)** Heatmap showing Average Nucleotide Identity (ANI) values from whole-genome comparisons between vineyard-derived *Bacillus* strains B4001, B4003, B4005, B4022, and B4023 from SAGWRI and their conspecific close relatives in GTDB.

The sequences based on the GTDB database were all grouped into *Bacillus velezensis* (strains B4003 and B4005) and *Bacillus*_sp018613535 (strains B4022, B4001 and B4023). Further interrogation of this clustering involved the creation of a dataset with *Bacillus* sensu stricto according to GTDB (Parks et al., 2022). Strains conspecific to our strains, as discussed above, were also included. This resulted in a dataset of 50 genomes, which included *Stenotrophomonas maltophilia* NCTC10257 as an outgroup. The UBCG phylogeny separated our strains into two lineages [Figure 1(I) and Supplementary Figure 1]. As expected, strains B4003 and B4005 grouped with *B. velezensis*, while three of the *Bacillus* strains (B4022, B4001, and B4023) consistently clustered with the undescribed strains, forming a well-supported monophyletic clade which have a placeholder “sp018613535” as-yet-undescribed *Bacillus* taxon on the GTDB database. The latter represents an undescribed putatively novel *Bacillus* lineage, which we will describe in our next study. Both groups, according to the UBCG phylogeny, received a high gene support index (GSI) of 92 (100%) for the *B. velezensis* cluster and 90 (98%) for the novel group (Figure 1(I) and Supplementary Figure 1). This analysis clearly indicates that the isolates are distinct from currently validly described *Bacillus* species, suggesting that they represent members of a previously uncharacterized taxonomic group.

The ANI values corroborated the phylogenetic clustering observed on the phylogeny above. In bacteria, the species threshold border is always determined upon an ANI value of 95%–96% (Palmer et al., 2020). Strains *Bacillus* spp. (strains B4022, B4001, and B4023) were conspecific with the undescribed strains with placeholder “sp018613535” sharing ANI values of >97%. Although within our strains, the ANI values were >99.0%. The remaining strains (B4003 and B4005) also showed >97% ANI values with *B. velezensis* and within themselves, having ANI values above 99.7% [Figure 1 (II)].

### 3.3 Pangenome and orthologs analyses

**Figure 2:**
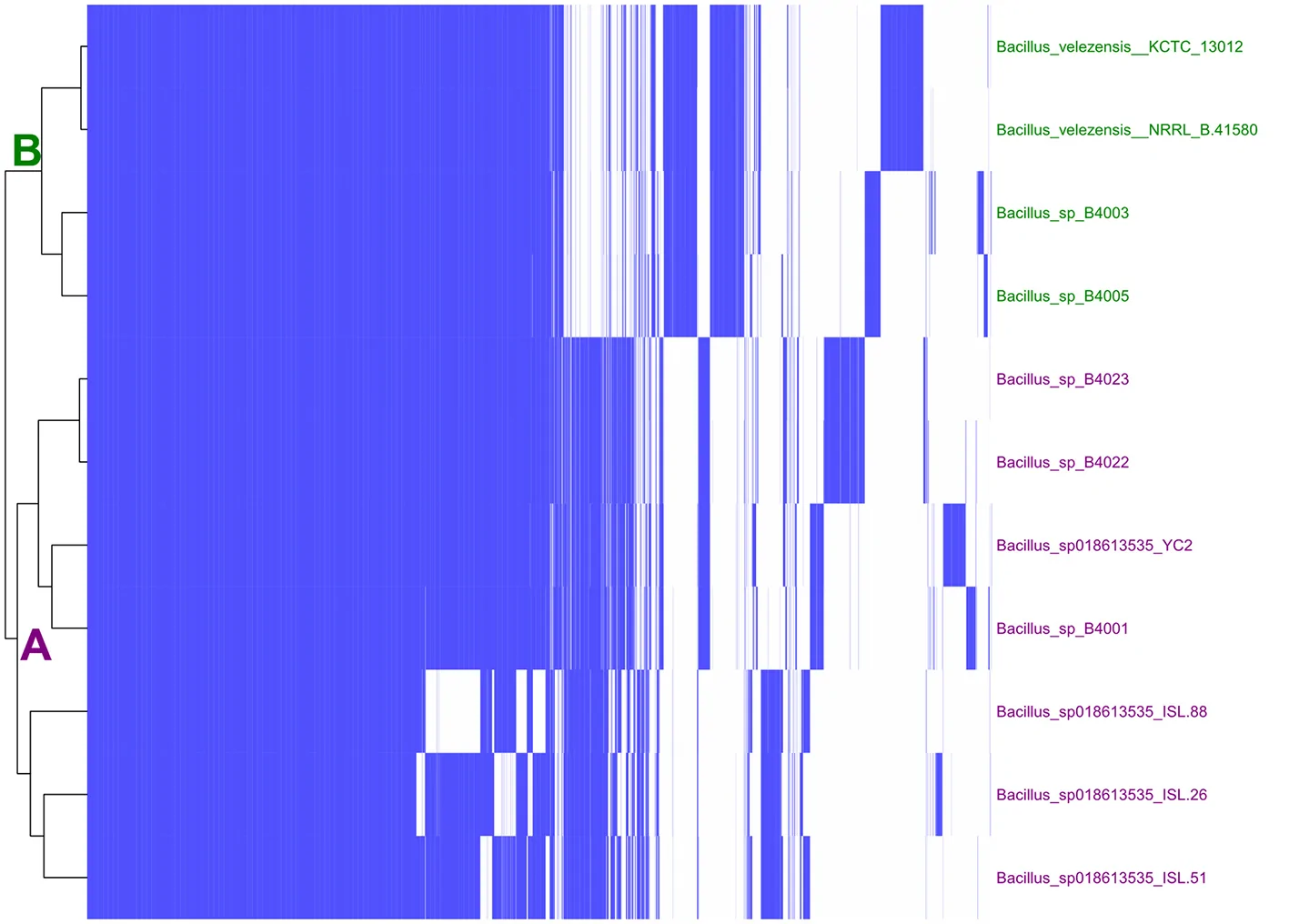
The pangenomic gene presence/absence plot of the *Bacillus* strains B4001, B4003, B4005, B4022, and B4023 from SAGWRI and their conspecific close relatives based on GTDB, showing the relative number of genes shared within and between the two clusters. Analyses were run using Roary at 80% BlastP identity. The node labels and color code represent the two clusters of the strains in this study as indicated in the phylogenetic tree in Figure 1.

The pan-genome analyses included our strains, two more type genomes of the *B. velezensis*, and three *Bacillus*_sp018613535 genomes. These were added for comparison and robustness purposes. From the analysis, a total of 5,735 genes were predicted, 2,088 of these represented the core genes, there were no soft core genes, 3,239 shell genes, and 408 cloud genes. The analysis revealed congruent pangenome presence/absence matrix compared to the phylogenetic clustering (Figure 2). The *B. velezensis* type strains showed similar clustering compared to the two strains in this study which also showed more similar clustering among themselves. On the other hand, the *Bacillus* sp018613535 clusters showed three of the selected strains from GTDB showing remarkable differences in the size of the core genome compared to others within the cluster (Figure 2). Two of the strains in this study were highly similar; the other one and one of the downloaded strains were comparable to others within the cluster (Figure 2). Overall, more differences were observed within the *Bacillus* sp018613535 cluster.

**Figure 3:**
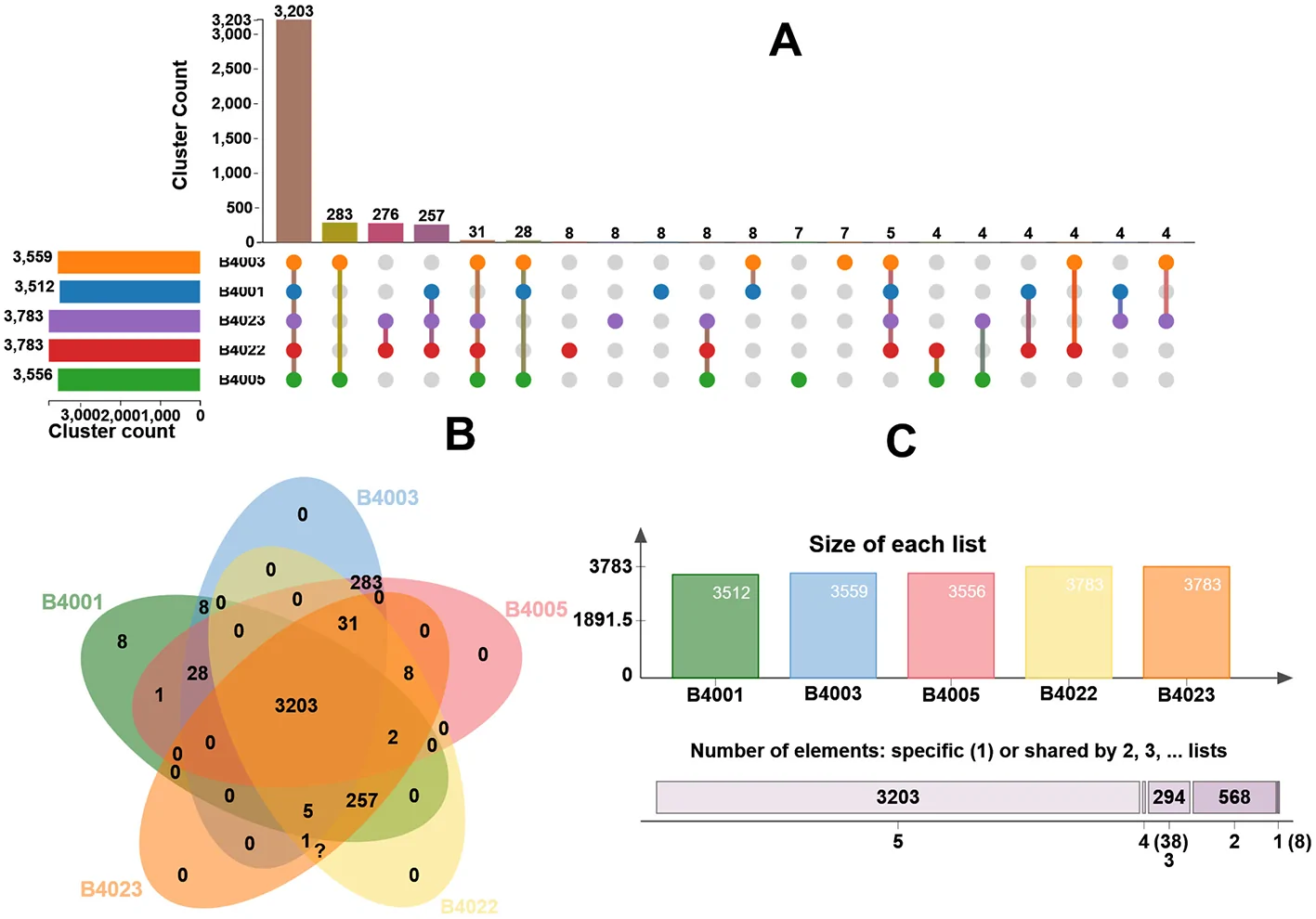
**(A)** Upset diagram showing relative distribution of number of shared clusters among the strains. **(B)** Avenn diagram showing the summarized shared clusters in all the strains and **(C)** a bar chart showing the number of clusters predicted in all the strains used in this study.

In terms of orthologs, 3559, 3512, 3783, 3783, and 3556 orthologs/clusters were observed for *B. velezensis* strain B4003, *Bacillus* spp. (strains B4001, B4023 and B4022), and *B. velezensis* (strain B4005, respectively (Figure 3). Only 3203 clusters were shared among all the strains, with *B. velezensis* (strains B4003 and B4005) sharing more (283). *Bacillus* spp. (strains B4023 and B4022) shared 276 orthologs (Figure 3), which is expected based on their phylogenetic grouping. Interestingly, strains B4001, B4023, and B4022 shared 257 orthologs. Thirty-one orthologs were shared among *B. velezensis* (strains B4003 and B4005), *Bacillus* spp. (strains B4023 and B4022), however, 28 shared orthologs were observed among *B. velezensis* (strains B4003 and B4005) and *Bacillus* sp. (strain B4001) (Figure 3).

**Figure 4:**
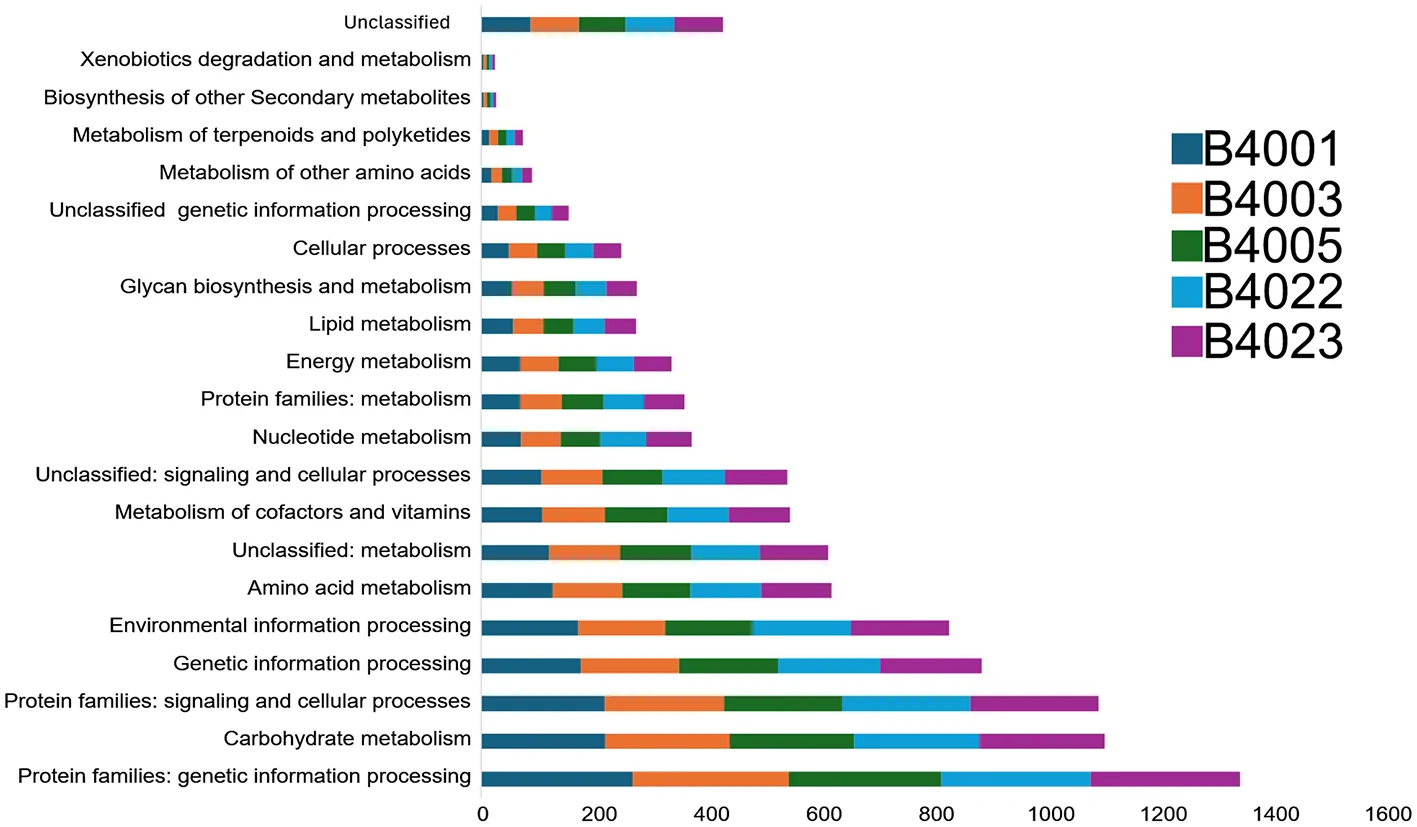
A stacked bar of the Ghost KOALA output of the strains in this study showing the distinct proportion of the functional categories based on the KEGG database.

The functional characterization, based on GhostKOALA analysis and run against the KEGG database, revealed differential functional categories among the strains (Figure 4). For instance, among the strains in the novel cluster for (*Bacillus* sp. strains B4001, B4022 and B4023) showed variations in genes involved in carbohydrate metabolism, nucleotide metabolism, and environmental information processing, among others. A similar trend was observed with the *B. velezensis* strains, where, although quite similar, differences were observed with genes involved in nucleotide metabolism and the unclassified metabolism (Figure 4).

### 3.4 Prediction of the biosynthesis gene clusters encoding secondary metabolite biosynthesis

**Figure 5:**
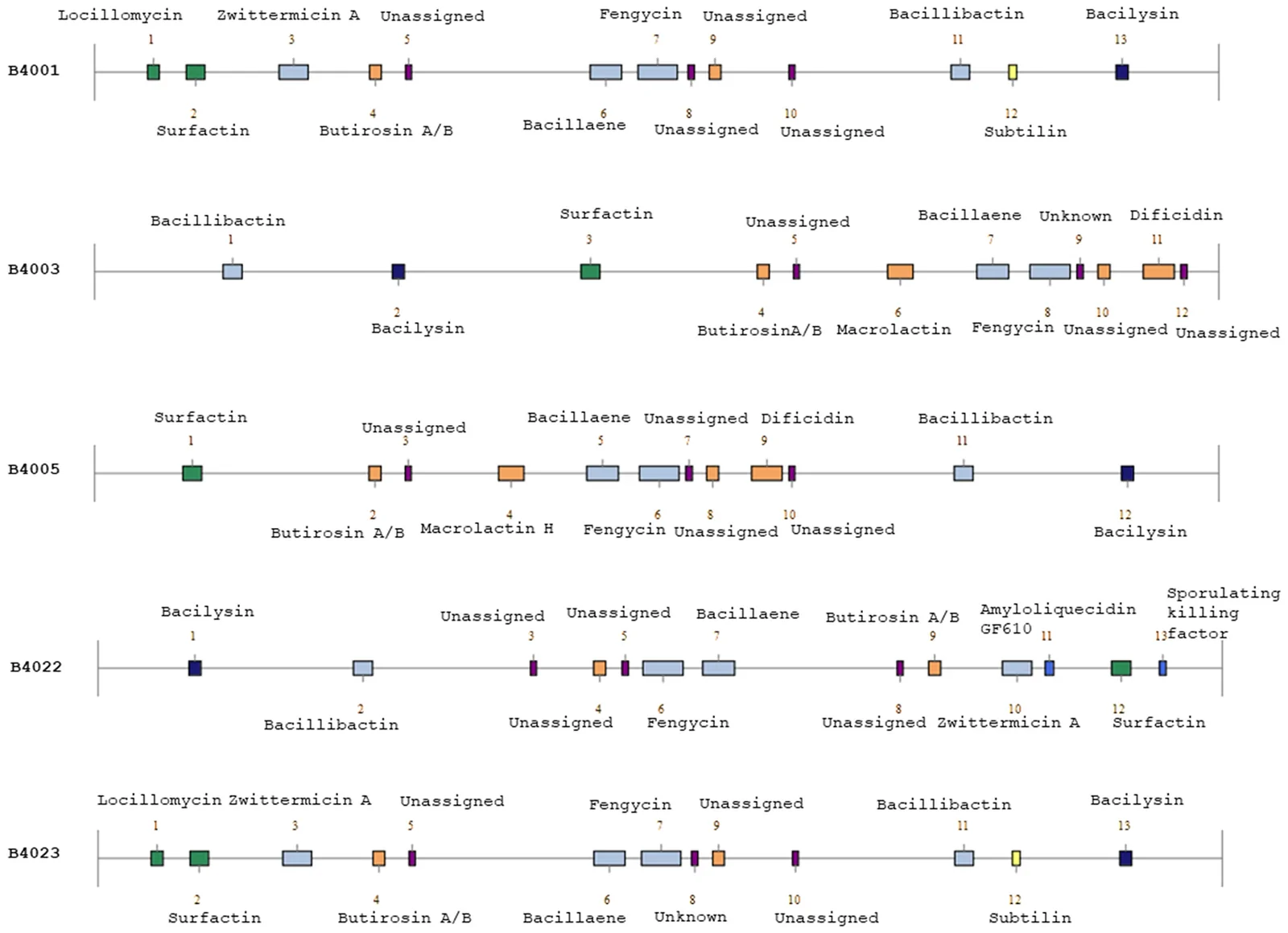
Comparisons of the locations of predicted secondary metabolite biosynthetic gene clusters in *Bacillus velezensis* (strains B4003 and B4005) and *Bacillus* sp. (strains B4022, B4001 and B4023).

Comparative antiSMASH analysis of genomes of the *Bacillus* strains revealed 17 distinct biosynthetic gene cluster (BCGs) regions across the dataset involved in secondary metabolite biosynthesis. Thirteen of these BCGs showed similarity to previously characterized biosynthetic gene clusters and were assigned putative secondary metabolites, while the remaining four were not assigned to known products and were classified as unknown (Table 3). The presence of these genes provided the genomic basis for the biosynthetic potential of the strains, which is consistent with but does not confirm the antagonistic activity against *B. cinerea* previously observed. Core biosynthesis gene clusters (BGCs) that encode enzymes for a specific biosynthetic pathway, typically producing secondary metabolites, including the non-ribosomal peptide synthetases (NRPSs) and NRPS-Hybrids, which produce lipopeptides, peptide antibiotics, siderophores, polyketide synthases (PKSs) (PKS-hybrids and betalactone) clusters, terpene and terpene-precursor clusters, ribosome-synthesized and post-translational modified peptides (RiPPs) (such as the lanthipeptide-class-I and II, cyclic lactone-autoinducer; santipeptide) and other biosynthetic cluster types (such as betalactone and NRP-metallophore) were predicted (Table 3). Of the BGCs regions predicted in all the genomes examined, nine putative BGCs were specifically found among *Bacillus* spp. (strains B4022, B4001 and B4023, respectively), while eight clusters were found among *Bacillus velezensis* (strains B4003 and B4005) encoding known biosynthetic gene clusters. The products and locations of the BGCs were compared as represented in Figure 5.

**Table 3:** Comparison of biosynthesis gene cluster regions of vineyard-isolated *Bacillus* spp. identified by antiSMASH.

Cluster	Type	Secondary metabolite	Functions	B4001	B4003	B4005	B4022	B4023
1	NRPS-like	locillomycinA/B/C	Antifungal, antibacterial	-	-		+	+
2	NRPS	Surfactin	Colonization, ISR, antiviral, antibacterial, antifungal	+	+	+	+	+
3	NRPS, t1PKS, HR-T2PKS	Zwittermicin A	Antibacterial and antifungal (broad-spectrum activity against prokaryotic and eukaryotic microorganisms)	+	-	-	+	+
4	PKS-like	Butirosin A/B	Antibacterial	+	+	+	+	+
5	Terpene	Unknown					-	
6	transAT-PKS, NRPS	Bacillaene	Antibacterial	+	+	+	+	+
7	NRPS-transAT-PKS, betalactone	Fengycin	ISR, antifungal	+	+	+	+	+
8	Terpene	Unknown			-	-	-	-
9	T3PKS	Unknown		-	-	-	-	-
10	Terpene-precursor	Unknown		-	-	-	-	-
11	Terpene-precursor, NRP-metallophore, NRPS	Bacillibactin	Antibacterial, antifungal, siderophore production	+	+	+	+	+
12	Lanthipeptide-class-i	Subtilin	Antibacterial (Gram-positive bacteria)	-	-	-	+	+
13	Lanthipeptide-class-ii, cyclic lactone-autoinducer	Amyloliquecidin GF610	Antibacterial (Gram-positive bacteria)	+	-	-	-	-
14	Sactipeptide	Sporulation killing factor	Antibacterial activity	+	-	-	-	-
15	transAT-PKS	Macrolactin	Antibacterial, antifungal, antiviral	-	+	+	-	-
16	transAT-PKS	Difficidin	Antibacterial		+	+		
17	Other	Bacilysin	Antibacterial, antifungal	+	+	+	+	+
	Total			9	8	8	9	9

NRPS, non-ribosomal peptide synthetase; PKS, polyketide synthase; t1PKS, type I polyketide synthase, T2PKS, type II polyketide synthase, transAT-PKS, trans-acyltransferase polyketide synthase; T3PKS, Type III polyketide synthase; NRP, non-ribosomal peptide; ISR, induced systemic resistance. Each row represents a predicted BCG identified by antiSMASH tool. Clusters annotated with multiple biosynthetic types (for instance, NRPS-transAT-PKS, betalactone) represent hybrid BCGs containing more than one biosynthetic domain and should be considered as a single BGC rather than separate clusters. The “unknown” represents predicted BGCs that were not confidently assigned to a characterized biosynthetic product based antiSMASH comparisons.

The gene clusters showed variation in their similarity compared with reference genomes. However, seven of the clusters in all the genomes displayed high homology, more than 70% similarity with known genes encoding the biosynthesis of secondary metabolites such as bacillaene, bacillibactin, fengycin, bacilysin, macrolactin, difficidin, amyloliquecidin, surfactin, subtilin, sporulating killing factor, and zwittermicin (Figure 5 and Supplementary Figures S2-S6). Six gene clusters encoding bacillibactin, bacillaene, bacilysin, butirosin, fengycin and surfactin were more prevalent and were found among all the genomes of the antagonistic *Bacillus* strains. Gene clusters encoding sporulating killing factor and amyloliquecidin GF610 were exclusively found in *Bacillus* sp. (strain B4001). However, locillomycin as well as subtilin gene clusters were found among *Bacillus* spp. (strains B4022 and B4023). Notably, the zwittermicin A BGC was found only in the genomes of all *Bacillus* spp. (strains B4022, B4001 and B4023), while macrolactin and difficidin clusters were found exclusively among *Bacillus velezensis* (strains B4003 and B4005).

## 4 Discussion

Research effort has been redirected toward the biological control of phytopathogens, driven by the need for sustainable, non-polluting crop production strategies. Soil microorganisms have been identified as integral components of the ecosystem, playing important roles in suppressing phytopathogens and enhancing agricultural productivity (Zhao et al., 2025). Among these, *Bacillus* spp. Particularly, members of the *B. subtilis* clade have been widely studied and demonstrated to inhibit diverse plant pathogens on a wide variety of fruit and vegetable crops (Adjei et al., 2025; Lastochkina et al., 2019; Wang et al., 2022). Whole-genome sequencing and comparative genomics have shown that species within the *B. subtilis* clade are genetically endowed to produce a plethora of antimicrobial compounds, which are not always accounted for in traditional analytical techniques. Consequently, genomic mining of newly identified strains is necessary to gain an in-depth understanding of their potential to produce secondary metabolites.

Multigene sequencing using 16S rRNA gene, *rpo*B, *rec*A, and *gyr*B had placed two of the strains (B4003 and B4005) as *B. velezensis* and the other three (B4001, B4022 and B4023) as *B. nakamurai* (Moremi et al., 2025). Whole-genome analysis revealed that all the strains exhibited genome features typical of the *Bacillus* genus, including %GC contents, a standard complement of RNA genes, and genome size similar to those reported earlier for *Bacillus* spp. (Mullins et al., 2020; Zhao et al., 2025). Moreover, phylogenetic classification, together with the ANI values above the 96% threshold for bacterial species (Palmer et al., 2020), further validated the classification of strains B4003 and B4005 as *B. velezensis*. Conversely, strains (B4022, B4001, and B4023), which were identified as *B. nakamurai* by 16S rRNA sequencing, only displayed ≈94% ANI compared to *B. nakamurai*. Since the ANI values are below the species demarcation threshold, the data suggest that these strains belong to a distinct species within the genus *Bacillus*. The strains consistently clustered with the formally undescribed lineage with placeholder “sp018613535” within the *Bacillus* genus and displayed an ANI >96%, suggesting that these three vineyard isolates belong to a putative novel lineage, indicating an important finding of this study. This placeholder designation represents a genome-based species identified through GTDB's standardized taxonomy but lacking a formal nomenclatural description of any validly named *Bacillus* lineage. This observation is an indication that the vineyard ecosystem may likely be a reservoir of novel *Bacillus* species. The placement of these strains in this cluster suggests that they are closely related to this provisional species and may belong to an emerging or currently uncharacterised *Bacillus* clade. Further studies, including digital DNA-DNA hybridization (dDDH) and broader phylogenomic placement, would be required to clarify whether these isolates represent strains within the same provisional species or potentially a distinct but related lineage.

Our analysis revealed a markedly different pan-genome architecture between the two *Bacillus* species, although several orthologous genes were shared across the genomes, consistent with previous studies, which demonstrated significant variations in gene content alongside a conserved core genome (Gayathri et al., 2025; Kim et al., 2017). *B. velezensis* strains clustered with reference strains, forming a distinct group, reflecting a well-conserved core genome typical of a coherent species cluster observed in *Bacillus* pan-genomic studies (Belbahri et al., 2017). Conversely, *Bacillus* spp. genomes exhibited more variations within their core genome, suggesting increased intra-group genomic diversity, consistent with previous observations that accessory and unique gene pools expand as additional genomes are included in pan-genome analyses (Asif et al., 2023). Orthologous cluster analysis revealed 3,203 shared gene content conserved across all strains, representing the core genome underpinning essential cellular functions. Accessory genes shared across phylogenetic groups suggest horizontal gene flow and functional convergence. The limited orthologs uniquely shared between species likely suggest that they contribute to strain-specific phenotypes, including specialized metabolism, environmental adaptation, stress responses and antimicrobial activity (Fu et al., 2021; Yang et al., 2025). Furthermore, functional characterization of the genes revealed notable differences in functional gene categories among the strains. Strains within the novel cluster (*Bacillus* spp. strains B4001, B4022 and B4023) exhibited greater variability in genes associated with carbohydrate metabolism, nucleotide metabolism, and environmental information processing, suggesting metabolic flexibility and adaptive responses to diverse ecological conditions. Similar functional diversification within closely related *Bacillus* lineages has been documented and is often linked to niche specialization and environmental adaptation (Das et al., 2024; Kanehisa et al., 2016). *B. velezensis* strains displayed a largely conserved functional profile. Differences were observed in nucleotide metabolism and unclassified metabolic categories, consistent with previous genomic studies indicating that accessory metabolic functions contribute to strain-specific phenotypes and ecological fitness in *Bacillus* species (Kim et al., 2017; Zeng et al., 2025).

Various secondary metabolites, including cyclic lipopeptides (such as iturins, fengycins, and surfactins), polyketides, and non-ribosomal synthetases, are secreted by *Bacillus* species (Zhao et al., 2025). Extracellular metabolites in these categories can disrupt fungal cell membranes, inhibit spore germination, and contribute significantly to antifungal activity (Zheng et al., 2024). We previously demonstrated that the cell-free extracts of *B. velezensis* B4005 contained surfactin and fengycin A and B, while strains B4001 and B4022 produced surfactin, fengycins A and B, as well as bacillomycin D (Moremi et al., 2025). These bioactive effects are encoded by specialized gene clusters. In the current study, cumulatively, thirteen BGCs, that showed similarity to previously characterized biosynthetic including bacillaene, bacillibactin, fengycin, bacilysin, macrolactin, difficidin, amyloliquecidin, surfactin, subtilin, sporulating killing factor, zwittermicin, bacillibactin, and butirosin, were predicted in all the genomes examined. Surprisingly, no gene clusters responsible for bacillomycin D biosynthesis were detected in strains B4001 and B4022. This discrepancy between the actual antimicrobial compounds identified and the presence of gene clusters was reported in a strain of *B. amyloliquefaciens* where C15-bacillomycin D was identified in the cell-free supernatant using ESI LC-MS/MS and proven to be responsible for the antifungal activity, although genome mining did not predict any iturin family or specifically bacillomycin biosynthesis gene cluster (Jin et al., 2025).

While several BGCs were common across all strains, the transAT-PKS type clusters encoding macrolactin and difficidin were unique to *B. velenzensis* strains B4003 and B4005. This is concordant with previous studies where these BGCs were highlighted as a common feature of *B. velezensis* genomes (Steinke et al., 2021). Macrolactin and difficidin, synthesized by the action of polyketide synthase, belong to different macrolide families and display different antimicrobial spectra. For instance, macrolactins can inhibit bacteria, fungi and viruses while difficidin is known for broad antibacterial activity (Aleti et al., 2015; Rabbee et al., 2019). The BGCs for zwittermicin A were unique to the *Bacillus* spp. strains B4001, B4022 and B4023, while those encoding the locillomycin and subtilin were only found in B4022 and B4023. The zwittermicin A BGC encodes a hybrid polyketide-non-ribosomal peptide pathway that produces the aminopolyol antibiotic known for its broad-spectrum antimicrobial activity against both bacterial and fungal phytopathogens (Stohl et al., 1999), while locillomycin clusters encode non-ribosomal peptide synthetase (NRPS) systems responsible for cyclic lipopeptides with broad antimicrobial effects (Gerst et al., 2022). Finally, *Bacillus* sp. strain B4001 was the only one harboring a sactipeptide-type gene cluster producing sporulation killing factor (SKF) and a lanthipeptide-class-II type cluster for biosynthesis of amyloliquecidin GF610. Sactipeptides, including SKF, represent ribosomally synthesized and post-translationally modified peptide (RiPP) pathways that produce thioether-linked antimicrobial cyclic peptides with a narrow spectrum of antimicrobial activity (Zhao and Kuipers, 2016). Moreover, SKF plays a key role in sporulation within the producing strain. The amyloliquecidin GF610 cluster encodes a two-component lantibiotic with potent antimicrobial activity against a range of gram-positive pathogens. Additionally, all results observed suggest that the *Bacillus* strains evaluated in this study possess promising attributes as potential natural antagonists of *B. cinerea*.

## 5 Conclusion

This study comparatively explored the genetic basis underlying the antagonistic attributes of vineyard-isolated *Bacillus* strains. An important finding of this study is that three of the vineyard isolates consistently grouped with a previously uncharacterized *Bacillus* species. The UBCG phylogeny and comparative pan-genome analysis of the *Bacillus velezensis* and *Bacillus* spp. revealed clear species-level differentiation alongside a conserved functional backbone, with the latter clustering with an undescribed putatively novel *Bacillus* lineage and relatively exhibiting greater accessory genome variability indicative of enhanced genomic plasticity. Genomic analysis revealed thirteen putative BGCs involved in secondary metabolite production, corroborating the antifungal activity of the strains. Orthologous cluster analysis further revealed 3,203 conserved across all strains, constituting a stable core genome that supports essential cellular and metabolic functions. In contrast, the presence of accessory genes shared across phylogenetic groups suggests horizontal gene transfer and functional convergence, likely contributing to adaptive traits such as antimicrobial activity and environmental resilience. Functional annotation revealed strain-specific enrichment in metabolic and environmental response pathways, emphasizing the ecological specialization and adaptive diversification. Collectively, these findings identified potentially undescribed *Bacillus* lineage and emphasize the dynamic interplay between genomic conservation and flexibility within the *Bacillus* genus. Further studies encompassing strain description, transcriptomics, metabolomics, and functional validation will be required to link the genomic potential with metabolic expression and to advance the application of these strains in sustainable agriculture and microbial-based disease management.

## Data Availability

The sequences generated in this study were deposited on the NCBI Genbank (https://www.ncbi.nlm.nih.gov/genbank) under BioProject accession number PRJNA1456001.
