# Supplementary material for: Genomic sequencing of vineyard-isolated Bacillus strains and comparison with closely related Bacillus species reveals a putative new lineage, distinct pan-genome architecture and secondary metabolism potential
Source: Front Microbiol. 2026 Aug 13;17:1871298. doi: 10.3389/fmicb.2026.1871298 (PMC13518137; doi:10.3389/fmicb.2026.1871298)
Supplement: Supplementary file 1 [file Supplementary_file_1.docx]

Supplementary Material

**Genomic sequencing of vineyard-isolated *Bacillus* strains and comparison with closely related *Bacillus* species reveals a putative new lineage, distinct pan-genome architecture and secondary metabolism potential**

**Adeoye J. Kayode^1†^, Edwin I. Wanjofu^2^ and Mathabatha Evodia Setati^1*^**

^1^South African Grape and Wine Research Institute, Stellenbosch University, Private Bag X1, Stellenbosch, 7599, South Africa.

^2^Department of Biochemistry, Genetics and Microbiology, University of Pretoria, Pretoria, South Africa.

*** Correspondence:**
Corresponding Author
[setati@sun.ac.za](mailto:setati@sun.ac.za)

†Current Address: Department of Biochemistry, Genetics and Microbiology, University of Pretoria, Pretoria, South Africa.

Table S1: PacBio sequencing summary

| Sample Name | Barcode Name | Polymerase Reads | Bases | Mean Read Length | Median base quality (MBQ) |
| --- | --- | --- | --- | --- | --- |
| B4022 | bc2065--bc2065 | 132.442 | 1,041,405,504 | 7.863 | 96.0 |
| B4003 | bc2066--bc2066 | 118.485 | 905,465,575 | 7.642 | 97.0 |
| B4005 | bc2067--bc2067 | 152.364 | 1,318,394,149 | 8.652 | 98.0 |
| B4001 | bc2068--bc2068 | 179.252 | 1,272,927,638 | 7.101 | 98.0 |
| B4023 | bc2070--bc2070 | 122.132 | 900,684,921 | 7.374 | 98.0 |

Fig. S1: Genome-based phylogenetic subtree of *Bacillus* spp. constructed using the UBCG pipeline, incorporating reference genomes retrieved from the GTDB database; *Stenotrophomonas maltophilia* NCTC10257 was used as an outgroup. The numbers on the nodes represent the Gene Support Index (GSI).

Figure S2: Gene clusters for biosynthesis of secondary metabolites in *Bacillus* sp. (strain B4022) with reference genomes.

Figure S3: Gene clusters for the biosynthesis of secondary metabolites in *Bacillus velezensis* (strain B4003) with reference genomes.

Figure S4: Gene clusters for the biosynthesis of secondary metabolites in *Bacillus velezensis* (strain B4005) with reference genomes.

Figure S5: Gene clusters for the biosynthesis of secondary metabolites in *Bacillus* sp. (strain B4001) with reference genomes.

Figure S6: Gene clusters for the biosynthesis of secondary metabolites in *Bacillus* sp. (strain B4023) with reference genomes.
